# The high frequency of *GJB2* gene mutation c.313_326del14 suggests its possible origin in ancestors of Lithuanian population

**DOI:** 10.1186/s12863-016-0354-9

**Published:** 2016-02-19

**Authors:** Violeta Mikstiene, Audrone Jakaitiene, Jekaterina Byckova, Egle Gradauskiene, Egle Preiksaitiene, Birute Burnyte, Birute Tumiene, Ausra Matuleviciene, Laima Ambrozaityte, Ingrida Uktveryte, Ingrida Domarkiene, Tautvydas Rancelis, Loreta Cimbalistiene, Eugenijus Lesinskas, Vaidutis Kucinskas, Algirdas Utkus

**Affiliations:** Department of Human and Medical Genetics, Faculty of Medicine, Vilnius University, Vilnius, Lithuania; Centre of Ear, Nose and Throat Diseases, Vilnius University Hospital Santariskiu clinics, Vilnius, Lithuania

**Keywords:** Non-syndromic sensorineural hearing loss, *GJB2* and *GJB6* genes, c.313_326del14 mutation, p.(Lys105Glyfs*5), Frequency of carriers of *GJB2* gene mutation in the Lithuanian population

## Abstract

**Background:**

Congenital hearing loss (CHL) is diagnosed in 1 – 2 newborns in 1000, genetic factors contribute to two thirds of CHL cases in industrialised countries. Mutations of the *GJB2* gene located in the *DFNB1* locus (13q11-12) are a major cause of CHL worldwide.

The aim of this cross-sectional study was to assess the contribution of the *DFNB1* locus containing the *GJB2* and *GJB6* genes in the development of early onset hearing loss in the affected group of participants, to determine the population-specific mutational profile and *DFNB1*-related HL burden in Lithuanian population.

**Methods:**

Clinical data were obtained from a collection of 158 affected participants (146 unrelated probands) with early onset non-syndromic HL. *GJB2* and *GJB6* gene sequencing and *GJB6* gene deletion testing were performed. The data of *GJB2* and *GJB6* gene sequencing in 98 participants in group of self-reported healthy Lithuanian inhabitants were analysed.

Statistic summary, homogeneity tests, and logistic regression analysis were used for the assessment of genotype-phenotype correlation.

**Results:**

Our findings show 57.5 % of affected participants with two pathogenic *GJB2 *gene mutations identified. The most prevalent *GJB2* mutations were c.35delG, p. (Gly12Valfs*2) (rs80338939) and c.313_326del14, p. (Lys105Glyfs*5) (rs111033253) with allele frequencies 64.7 % and 28.3 % respectively. *GJB6* gene mutations were not identified in the affected group of participants. The statistical analysis revealed significant differences between *GJB2*(−) and *GJB2*(+) groups in disease severity (*p* = 0.001), and family history (*p* = 0.01). The probability of identification of *GJB2* mutations in patients with various HL characteristics was estimated. The carrier rate of *GJB2* gene mutations – 7.1 % (~1 in 14) was identified in the group of healthy participants and a high frequency of *GJB2*-related hearing loss was estimated in our population.

**Discussion:**

The results show a very high proportion of GJB2-positive individuals in the research group affected with sensorineural HL. The allele frequency of c.35delG mutation (64.7 %) is consistent with many previously published studies in groups of affected individuals of Caucasian populations. The high frequency of the c.313_326del14 (28.3 % of pathogenic alleles) mutation in affected group of participants was an unexpected finding in our study suggesting not only a high frequency of carriers of this mutation in our population but also its possible origin in Lithuanian ancestors. The high frequency of carriers of the c.313_326del14 mutation in the entire Lithuanian population is supported by it being identified twice in the ethnic Lithuanian group of healthy participants (a frequency 2.0 % of carriers in the study group).

**Conclusion:**

Analysis of the allele frequency of *GJB2* gene mutations revealed a high proportion of c. 313_326del14 (rs111033253) mutations in the *GJB2*-positive group suggesting its possible origin in Lithuanian forebears. The high frequency of carriers of *GJB2* gene mutations in the group of healthy participants corresponds to the substantial frequency of *GJB2*-associated HL in Lithuania. The observations of the study indicate the significant contribution of *GJB2* gene mutations to the pathogenesis of the disorder in the Lithuanian population and will contribute to introducing principles to predict the characteristics of the disease in patients.

**Electronic supplementary material:**

The online version of this article (doi:10.1186/s12863-016-0354-9) contains supplementary material, which is available to authorized users.

## Background

Congenital hearing loss (CHL) is a disease of considerable concern in medicine nowadays. It is one of the most common conditions and is diagnosed in 1 – 2 of 1000 newborns [1]. The incidence rises to 3.5 of 1000 before adolescence [2]. The disorder is highly heterogeneous; every population has a unique HL etiologic profile dependent on ethnic, geographic, social and medical factors. Genetic factors contribute to up to two thirds of CHL cases in industrialized countries [3]. Most cases, about 70 %, have non-syndromic hearing loss and about 30 % represent syndromic deafness [4]. The remaining one-third of cases can be ascribed to environmental and unidentified genetic factors.

At least 400 and over 150 genetic loci are associated with syndromic and non-syndromic hearing loss respectively [5, 6]. The inheritance of the disorder may be autosomal dominant, autosomal recessive, X recessive and mitochondrial.

Pathogenic mutations in the *DFNB1* (Deafness) locus (13q11-12) containing *GJB2* (NM_004004.5) and *GJB6* (NM_001110219.2) genes are the most common cause of non-syndromic sensorineural hereditary hearing loss worldwide [7]. Results of the analysis of the *DFNB1* locus in patients in different populations demonstrate the leading role of that pathogenic changes in the *GJB2* and *GJB6* genes, which account from 10 to 40 % of cases, have in etiologic profile of sensorineural HL [8].

To date more than 300 pathogenic mutations of the *GJB2* gene and over 20 pathogenic mutations including gross del(*GJB6*-D13S1830) and del(*GJB6*-D13S1854) in the *GJB6* gene have been determined leading to development of sensorineural HL [9].

*GJB2* and *GJB6* genes undergo coordinated transcription, and their major expressing organs are cochlea, placenta, hepatocytes, skin, pancreas, kidney and intestine (*GJB2* gene), and astrocytes, cochlea (*GJB6* gene) [10]. Connexin 26, a 226 amino acid protein encoded by the *GJB2* gene (OMIM* 121011) and connexin 30, 261 amino acid protein encoded by the *GJB6* gene (OMIM* 604418), form connexons. These homo- or heteromeric structures compose gap junctions and are essential in the transport of K^+^, Ca^2+^ ions, IP_3_, and other small molecules between many cells in an organism including the supporting cells in the inner ear, and provide a direct pathway of communication for intercellular electrical and chemical signaling. The altered recycling of K^+^ to the endolymph of the cochlea disturbs the repolarization of the hair cell membrane and formation of an auditory nerve impulse [11].

The disease characteristics (severity, symmetry, age of onset, etc.) of sensorineural HL linked with *DFNB1* vary and were shown to be dependent on the *GJB2* and/or *GJB6* genotype and the nature of the pathogenic mutation [12]. Most *GJB2* and *GJB6* mutations are associated with autosomal recessive non-syndromic hearing loss, but several dominant mutations, causing sensorineural HL or syndromic hearing loss (KID (MIM 148210), Vohwinkel (MIM 124500) syndromes, palmoplantar keratoderma (MIM 148350)) have been described [13].

Analysis of the mutational spectrum in the *DFNB1* locus, genotype – phenotype correlation, and analysis of the burden of *DFNB1*-related hearing loss have been performed in many countries. The aim of our cross-sectional study was to assess the contribution of *GJB2* and *GJB6* gene mutations to the development of early onset hearing loss and determine the mutational profile in the affected group of participants in the Lithuanian population and to analyse the burden of *GJB2* and *GJB6* gene mutations in our population, adding missing puzzle piece to the genetics of congenital hearing loss.

## Methods

### Study design

We performed cross-sectional study using data of the two observational projects: DEAFGEN and LITGEN. One of the aims of DEAFGEN is to identify pathogenic mutations of known genes associated with hereditary hearing loss and characterize their phenotypes. The aim of the LITGEN project was to perform wide scale genomic studies of the population of Lithuania and to identify genomic regions of hypothetical Lithuanian which are significant for health. Data and results of whole genome, whole exome and genome-wide genotyping becomes the background for the reference genome of the population of Lithuania and a variety of studies related with monogenic and complex diseases in the population of Lithuania.

We enrolled two groups of participants (DEAFGEN group: individuals affected with early onset HL and LITGEN group: individuals of ethnic Lithuanian population) in the current study, and collected data and venous blood samples for the clinical and genetic analysis. Population-specific *DFNB1* locus mutation profile was determined in both groups of participants, genotype – phenotype correlation analysis performed in affected group of participants (DEAFGEN), and burden of *DFNB1*-related HL in our population assessed (LITGEN).

### Recruitment of participants with non-syndromic sensorineural HL (DEAFGEN project)

Patients affected with early onset (before 5 years of age) non-syndromic hearing loss referred to the Vilnius University Hospital Santariskiu Clinics Centre for Medical Genetics and Centre of Ear, Nose and Throat from 2010 to 2015 were enrolled in this study.

Demographic data and medical records were obtained and physical examination and genealogy analysis were performed. In the presence of several affected relatives in the family, only one (randomly chosen) was recruited to the study to avoid bias of analysis.

Subgroups of affected participants were formed according to the results of the analysis of disease severity, symmetry and three generation genealogy. The participant was assigned to the positive genealogy subgroup if at least one relative with early onset hearing loss was determined in the family or negative genealogy subgroup – if the case was apparently sporadic.

### Clinical evaluation of the affected group

All participants in the study were assessed in accordance with age-specific specialised audiological evaluations. Pure-tone audiometry was obtained when possible, with the use of a diagnostic audiometer in a soundproof booth, in accordance with ISO standards. The threshold values in decibels (dB) for 0.5, 1, 2 and 4 kHz were averaged for both ears (pure-tone average PTA). In cases without pure-tone audiometry, the threshold of the wave V of the click-evoked auditory brainstem responses ABR or auditory steady state response ASSR were used to calculate the hearing level. The definition of the degree and type of HI was based on the most recent audiogram available. The degree of HL was classified according to the PTA (or extrapolated auditory brainstem responses value) as mild (21–40 dB), moderate (41–70 dB), severe (71–90 dB), or profound (>90 dB). The severity of deafness was defined by the degree of hearing loss in the better ear. Asymmetry was defined if the PTA between ears revealed the difference of 15 dB or greater.

Venous blood samples and written informed consent forms of affected participants or their parents (in the case of minors under the age of 16 years) were collected for the ‘The genomics of congenital/hereditary hearing loss: implication in disease pathogenesis, influence to phenotypic expression and treatment efficiency’ (acronym: DEAFGEN) project. The approval to conduct the DEAFGEN project was provided by the Vilnius Regional Research Ethics Committee.

### Recruitment of ethnic Lithuanian population group of healthy individuals (LITGEN project)

The group of healthy participants consisted of 98 unrelated adult individuals. This group represents the pure ethnic Lithuanian population due to the strict criteria of the enrolment conferring the uniqueness of this group: all self-reported healthy study participants indicated at least three generations of Lithuanian ethnicity and residency in the same ethno-linguistic region.

The data, venous blood samples and written informed consent forms were collected from individuals (trios) who were invited to the primary healthcare centers in the different regions of Lithuania (West, North, South Zemaitija and West, East, South Aukstaitija) in the period 2011–2013 for the ‘Genetic diversity of the population of Lithuania and changes of its genetic structure related with evolution and common diseases’ (acronym: LITGEN) project. The approval to conduct the LITGEN project was provided by the Vilnius Regional Research Ethics Committee. No follow-up or exposure was performed.

### Genetic analysis in affected group of participants

Whole genomic DNA was extracted from peripheral blood following the standard phenol-chloroform extraction protocol.

Polymerase chain reactions (PCR) of coding sequence and sequences flanking splicing site mutation c. − 23 + 1G > A (rs80338940) of the *GJB2* and *GJB6* genes were performed using specific primers designed with Primer Blast (NCBI) software [14] (see Additional file 1). Both strands of PCR products were sequenced with BigDye® Terminator v3.1 Cycle Sequencing Kit (Applied Biosystems, USA). Capillary electrophoresis was carried out with 3130xL Genetic Analyser (Applied Biosystems, USA). Fluorescent signals were analysed with Sequence Analysis v5.1 software (Applied Biosystems, USA). The sequences obtained were aligned with the reference sequence of the *GJB2* (NCBI NM_004004.5) gene. The sequence variants were analyzed in the Human Gene Mutation Database [15] and Connexin Deafness Homepage [16] Segregation analysis was performed by sequencing the *GJB2* gene to the parents of the affected participants.

The multiplex PCR assay designed by del Castillo [17] was used to detect the del(*GJB6*-D13S1830) and del(*GJB6*-D13S1854) mutations in the group of affected participants if *GJB2* mutations were not identified or only one heterozygous *GJB2* mutation was identified.

The frequencies of the *DFNB1* mutations were defined and inactivating as well as non-inactivating mutations assessed. After the genetic testing, three major subgroups *GJB2*(+), *GJB2*(+/−) and *GJB2*(−) were formed to perform the statistical analysis of genotype – phenotype correlation. *GJB2*(+) subgroup was divided into classes of genotypes according to possession of inactivating (frameshift) or non-inactivating (missense) mutation to determine their different impact on the characteristics of the disease.

### Genetic analysis in group of ethnic Lithuanian population

Genomic DNA was extracted from blood using the phenol–chloroform extraction method or automated nucleic acid purification using paramagnetic particles (TECAN Freedom EVO® 200, Tecan Schweiz AG, Switzerland). Next-generation exome sequencing after in-solution capture enrichment (TargetSeq™, Life Technologies or SureSelect, Agilent) with an average of a 40-fold coverage was performed at the Department of Human and Medical Genetics, Faculty of Medicine, Vilnius University with the use of a 5500 SOLiD™ Sequencer according to the optimised manufacturer’s protocols. Sequence alignment and secondary and tertiary analysis performed using LifeScope™ Genomic Analysis Software v2.5. The Genome Analysis Toolkit’s (GATK) CombineVariants tool [18, 19] was used to combine all identified genomic variants from 98 individuals into single VCF file. The genomic variants identified were annotated using the Annovar v.2015mar22 [20] program. Each identified *GJB2* and *GJB6* gene variant was checked by analysing individuals’ BAM files using the visualisation tool Integrative Genomics Viewer (IGV) [21].

### Statistical analysis

Hearing loss characteristics (severity and symmetry), family history and allele frequencies were treated as outcome variables in the analysis. Results of genetic testing (*GJB2* and *GJB6* genotypes) were treated as predictors.

Categorical variables were expressed as absolute numbers and percentages. The binomial exact test was applied to calculate a confidence interval 95 % for a proportion. The homogeneity hypothesis between two variables was tested using Pearson’s chi-square. Logistic regression analysis was conducted to assess the impact of *GJB2* gene mutations on HL severity and positive family history. P-values less than 0.05 were considered statistically significant. The statistical software package R (version 3.2.1) was used to obtain the results. G*Power (version 3.1) was used for *post hoc* power analysis of the test employed.

## Results

### Affected group of participants

One hundred fifty-eight participants (146 unrelated probands), 77 female and 81 male with non-syndromic early onset (all children aged under five) hearing loss were enrolled in the affected group of the research. The anonymized data of *GJB2* gene genotypes are provided in the Dataset of the group of affected individuals (see Additional file 2, the number given to each participant does not enable his identification).

### Results of clinical evaluation in affected group of participants

According to clinical evaluation profound, severe, moderate and mild hearing loss was identified in 85 (53.8 %), 24 (15.2 %), 37(23.4 %), and 12(7.6 %) of the affected participants respectively.

One hundred forty (88.6 %) individuals suffered from symmetrical hearing loss and 18 (11.4 %) had non-symmetric HL.

Genealogy analysis revealed 61 (41.8 %) unrelated participants with positive family history of early onset hearing loss and 85 (58.2 %) individuals without affected family members (Table 1).

**Table 1:** The results of clinical evaluation

Feature	Type	Counts	%
Severity	Mild	12	7.6
	Moderate	37	23.4
	Severe	24	15.2
	Profound	85	53.8
Symmetry	Symmetric	140	88.6
	Non-symmetric	18	11.4
Genealogy	Positive	61	41.8
	Negative	85	58.2

### Research group of ethnic Lithuanian population

The group of the ethnic Lithuanian population consisted of 98 unrelated, self-reported healthy individuals (49 female and 49 male participants). The anonymised data with *GJB2* and *GJB6* genes genotypes are provided in the Dataset of the group of ethnic Lithuanian population (see Additional file 3, the number given to each participant does not enable his identification).

### Power analysis

#### Affected group of participants

The *post hoc* power analysis was performed for all the tests in the study. Calculated empirical effect size ranged from medium to large. The empirical power of the tests was above 0.8. We present values for the empirical effect size and power in corresponding Tables.

#### Ethnic Lithuanian group

Assuming medium effect size for the binomial exact test and having sample size of 98, the calculated power is above 0.8.

### *GJB2* gene mutation spectrum in affected group of participants

**Fig 1:**
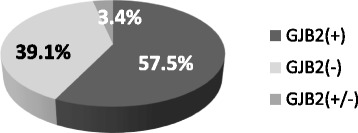
Results of *GJB2* gene testing

*GJB2* gene coding sequence analysis revealed 2 pathogenic mutations in homozygous or compound heterozygous state in 84 (57.5 %) affected unrelated participants, 5 (3.4 %) individuals had 1 mutation in heterozygous state and 57 (39.1 %) unrelated participants had no causative *GJB2* gene mutations (Fig. 1).

A total of seven different pathogenic mutations (frameshift and missense) were identified in the affected group of participants (Table 2).

**Table 2:** Allele frequencies of pathogenic *GJB2* gene mutations in the affected group of unrelated participants

Pathogenic *GJB2* gene mutation	Count	Allele frequency (%)	Prediction by *in silico* computational analysis
c.35delG, p.(Gly12Valfs*2), rs80338939	112	64.7	**MutationTaster2** Disease causing
c.313_326del14 p.(Lys105Glyfs*5), rs111033253	49	28.3	**MutationTaster2** Disease causing
c.269 T > C p.(Leu90Pro), rs8033894	4	2.3	**Sift** Damaging (score 0.000)
			**Polyphen-2** Probably damaging (score 1.0)
			**MutationTaster2** Disease causing
c.101 T > C p.(Met34Thr), rs35887622	4	2.3	**Sift** Damaging (score 0.027)
			**Polyphen-2** Benign (score 0.083)
			**MutationTaster2** Disease causing
c.167delT p.(Leu56Argfs*26), rs80338942	2	1.2	**MutationTaster2** Disease causing
c.109G > A p.(Val37Ile), rs72474224	1	0.6	**Sift** Tolerated (score 0.717)
			**Polyphen-2** Probably damaging (score 1.0)
			**MutationTaster2** Disease causing
c.379C > T p.(Arg127Cys), rs727503066	1	0.6	**Sift** Damaging (score 0.0)
			**Polyphen-2** Benign (score 0.423)
			**MutationTaster2** Disease causing
Total	173	100.0	

The most prevalent *GJB2* gene mutation in our study group was c.35delG, p.(Gly12Valfs*2) (rs80338939), which accounts for 64.7 % of pathogenic alleles. This change was identified in a homozygous state in 37 unrelated affected individuals (44.0 % of the *GJB2*-positive group), also in a heterozygous state with c.313_326del14, p.(Lys105Glyfs*5) (rs111033253) in 28 participants (33.3 % of the *GJB2*-positive group) and with other mutations in 8 participants (9.5 % in *GJB2*-positive group) (Table 3).

**Table 3:** Genotype distribution of pathogenic *GJB2* gene mutations in the *GJB2*-positive subgroup of affected participants

*GJB2* genotype	Profound	Severe	Moderate	Mild	Total count	%
c.[35delG];[35delG]	30	5	2	-	37	44.0
c.[35delG];[313_326del14]	20	4	4	0	28	33.3
c.[313_326del14];[313_326del14]	5	1	3	0	9	10.7
c.[35delG];[269 T > C]	1	-	-	2	3	3.6
c.[35delG];[101 T > C]	-	-	-	2	2	2.4
c.[35delG];[167delT]	-	-	1	-	1	1.2
c.[35delG];[379C > T]	1	-	-	-	1	1.2
c.[35delG];[109G > A]	-	-	1	-	1	1.2
c.[269 T > C];[313_326del14]	-	-	-	1	1	1.2
c.[c.101 T > C];[313_326del14]	-	-	-	1	1	1.2
Total *GJB2* (+)	57	10	11	6	84	100.0

The second most frequent mutation in the group of affected participants was c.313_326del14, p.(Lys105Glyfs*5) (rs111033253) with a frequency 28.3 % of pathogenic alleles. Nine unrelated affected individuals (10.7 % of the *GJB2*-positive group) possess the mutation in homozygous state, 28 individuals (33.3 %) – possess it in a heterozygous state with c.35delG, p.(Gly12Valfs*2) (rs80338939) and 2 participants possess it in compound with other mutation (2.4 %).

Other pathogenic mutations: c.269 T > C, p.(Leu90Pro) (rs8033894), c.101 T > C, p.(Met34Thr) (rs35887622), and c.109G > A, p.(Val37Ile) (rs72474224) were much rarer – each accounted for only up to 2.3 % of pathogenic alleles and were in a heterozygous state with either of the two most frequent mutations. The variant of unknown significance, c.379C > T, p.Arg127Cys (rs727503066), was assigned to pathogenic mutations due to a previous publication [22] and our observations (see *Discussion*).

### *GJB2* gene mutation spectrum in ethnic Lithuanian population group

Data of *GJB2* gene coding exon sequencing of 98 unrelated participants were analysed.

The results revealed the heterozygous state of *GJB2* mutations in 7 DNA samples (7.14 %) showing that approximately 1 in 14 individuals in the Lithuanian population is a carrier of the *GJB2* gene mutation (Table 4). Three healthy study participants had the c.101 T > C, p.(Met34Thr) (rs35887622) mutation (3.1 %), and the mutations c.313_326del14, p.(Lys105Glyfs*5) (rs111033253) and c.35delG, p.(Gly12Valfs*2) (rs80338939) were discovered in a heterozygous state with 2.0 % and 1.0 % carrier frequencies respectively. A novel, previously undescribed truncating mutation c.206delT (p.Phe69Serfs*13) was identified in one participant (genotype frequency 1.0 %).

**Table 4:** Allele frequencies and carrier frequencies of the pathogenic *GJB2* gene mutations in the healthy group of Lithuanian population

Mutation	Count	Allele frequency, %	Carrier frequency, % (95 % CI)
c.101 T > C (p.Met34Thr) rs35887622	3	1.5	3.1 (0.6−8.7)
c.313_326del14 (p.Lys105Glyfs*5) rs111033253	2	1.0	2.0 (0.3−7.2)
c.35delG (p.(Gly12Valfs*2)) rs80338939	1	0.5	1.0 (0.1−5.6)
c.206delT (p.Phe69Serfs*13)	1	0.5	1.0 (0.1−5.6)
Total			7.1(2.9−14.2)

According to the frequency of carriers of the *GJB2* gene mutation in healthy group of our study (7.1 % or ~1 in 14) we estimated the rate of *GJB2*-related sensorineural HL in Lithuanian population – at approximately 1 in 772 in case of non-assortative marriages.

### Results of testing *GJB6* gene point mutations and gross deletions

*GJB6* gene point pathogenic mutations and deletions del(*GJB6*-D13S1830) and del(*GJB6*-D13S1854) were not identified in our affected group of participants.

The *GJB6* gene mutation c.428G > A, p.(Arg143Gln) (rs201783640) was identified in a heterozygous state in one DNA sample in the study group of healthy individuals (carrier frequency ~1.0 %). The change was evaluated by *in silico* analysis: SIFT predicted that amino acid substitution likely affects protein function with score 0.003, Polyphen2 predicted probably damaging with score 0.998[23]; MutationTaster2 predicted the change to be disease causing.

### Genotype – phenotype correlation analysis

To analyse genotype – phenotype correlation the group of affected participants was divided into three major subgroups according to the results of *GJB2* gene sequencing. The *GJB2*-positive subgroup consisted of individuals with two (homozygous or compound heterozygous) mutations identified, the *GJB2*(+/−) subgroup consisted of individuals with one heterozygous *GJB2* gene mutation identified, and *GJB2*-negative subgroup consisted of affected participants with no pathogenic *GJB2* gene mutations identified. The *GJB2*(+) and *GJB2*(−) subgroups were compared with each other to determine the difference in disease severity, symmetry, and family history.

Data of five individuals of the *GJB2*(+/−) subgroup with a single autosomal recessive mutation was not included in the genotype – phenotype correlation analysis to avoid bias of ascertainment.

Homogeneity tests were employed to evaluate the impact of the *DFNB1* genetic locus on the hearing loss phenotype in the affected group of participants.

**Fig 2:**
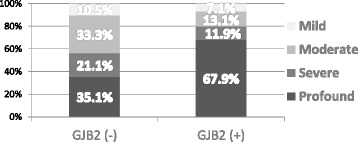
Distribution of HL severity in *GJB2*(+) and *GJB2*(−) subgroups of affected group of participants

Our study results indicate that the severity of hearing loss differs statistically significantly between the *GJB2*(+) and *GJB2*(−) subgroups, *p* = 0.001 (Table 5, Fig. 2). Profound HL dominates in the *GJB2*-positive subgroup while moderate and mild HL is more common in the *GJB2*-negative subgroup.

**Table 5:** Distribution of HL severity of *GJB2*(−) and *GJB2*(+) subgroups

	Profound HL	Severe HL	Moderate HL	Mild HL	Total
*GJB2* (−)	20	12	19	6	57
*GJB2* (*+*)	57	10	11	6	84
Total	77	22	30	12	141
Pearson Chi-Square 15.5			*p* = 0.001		
Empirical effect size w = 0.7			Empirical power = 1.0

To compare the influence of inactivating (frameshift) and non-inactivating (missense) *GJB2* gene mutations on the characteristics of the disease, the *GJB2*(+) subgroup was divided into two classes of genotypes I/I and I/N (I/I two inactivating (frameshift) mutations of the *GJB2* gene identified; I/N inactivating and non-inactivating (missense) mutations of the *GJB2* gene in compound heterozygosity identified).

**Fig 3:**
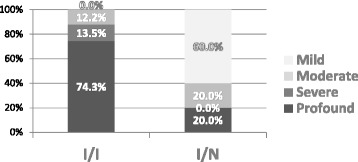
Distribution of degree of HL in I/I and I/N classes of *GJB2* (+) subgroup

A statistically significant difference in the distribution of HL severity in the classes of the *GJB2*(+) group was observed, *p* = 8.005x10^−11^ (Table 6, Fig. 3), with profound HL prevailing in the I/I subgroup and mild HL in the I/N subgroup.

**Table 6:** Distribution of degree of HL in I/I and I/N classes of *GJB2* (+) subgroup

Mutation type	Profound	Severe	Moderate	Mild	Total
	HL	HL	HL	HL	
I/I	55	10	9	0	74
I/N	2	0	2	6	10
Total	57	10	11	6	84
Pearson Chi-Square 50.0				*p* = 8.005x10^−11^	
Empirical effect size w = 1.6				Empirical power = 1.0

**Fig 4:**
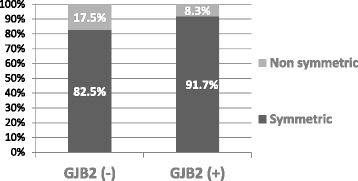
Distribution of HL symmetry in *GJB2*(+) and *GJB2*(+) subgroups

The influence of *GJB2* gene mutations on symmetry of hearing loss was also analysed in affected group of individuals. The difference between *GJB2*- positive and *GJB2*-negative subgroups was not statistically significant, *p* = 0.099 (Table 7, Fig. 4).

**Table 7:** Distribution of HL symmetry in *GJB2*(+) and *GJB2*(−) subgroups

	Non symmetric	Symmetric	Total
*GJB2* (−)	10	47	57
*GJB2* (+)	7	77	84
Total	17	124	141
Pearson Chi-Square 2.7			*p* = 0.099
Empirical effect size w = 0.3			Empirical power = 0.9

The genealogies of three generation of affected group of individuals were analyzed to assess the heredity of hearing loss. When more than one affected individuals with early onset hearing loss was present in the family assignment to the positive family history was made.

**Fig 5:**
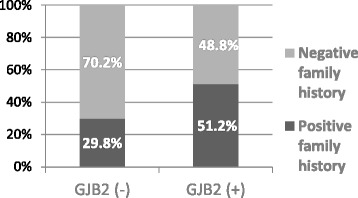
Distribution of genealogy types in *GJB2*(+) and *GJB2*(−) subgroups

The comparison of *GJB2*(+) and *GJB2*(−) subgroups showed a statistically significant difference between the subgroups, *p* = 0.012, indicating a more frequent positive family history in the *GJB2* (+) subgroup (Table 8, Fig. 5).

**Table 8:** Distribution of genealogy types in *GJB2*(+) and *GJB2*(−) subgroups

	Positive family history	Negative family history	Total
*GJB2* (−)	17	40	57
*GJB2* (+)	43	41	84
Total	60	81	141
Pearson Chi-Square 6.3			*p* = 0.012
Empirical effect size w = 0.5			Empirical power = 1.0

We applied logistic regression analysis to evaluate the chances of identifying the two *GJB2* gene mutations if profound/severe hearing loss vs moderate/mild hearing loss was diagnosed. OR 3.1 (95 % CI 1.5 – 6.6; *p* = 0.003) was calculated in our study group of affected individuals meaning the probability of having *GJB2* mutations is 3.1 times higher in case of profound or severe HL (Table 9).

We also estimated the probability of identifying *GJB2* gene mutations in affected individuals with a positive family history in our population. The results indicate that the chances are 2.5 times higher than they are in individuals with a negative family history, *p* = 0.013 (Table 9).

**Table 9:** Probabilities of identification of *GJB2* gene mutations to affected individual

HL characteristics	OR (95 % CI)	p	Empirical power
Profound/severe *vs* moderate/mild HL	3.1 (1.5 − 6.6)	0.003	0.9
Positive family history *vs* negative family history	2.5 (1.2 − 5.1)	0.013	0.8

## Discussion

Hearing loss is considered to be a very heterogeneous disorder. Although many genes have been associated with hearing loss, mutations in the *DFNB1* locus are to be the most frequent cause of autosomal recessive hearing loss and routine sequencing of the *GJB2* gene and testing of *GJB6* gene deletions are recommended in EMQN best practice guidelines [24].

Our study aimed to analyse the impact of *DFNB1* locus mutations on the development of early onset hearing loss in an affected group of participants in the Lithuanian population. This group consists of 146 unrelated individuals. Our analysis describes a substantial part: about 0.25–0.5 per cent of deaf people in Lithuania. The results show a very high proportion of *GJB2*-positive individuals (57.5 %) in the research group affected with sensorineural HL compared with other Caucasian populations representing an adequate selection of patients for genetic testing by referring physicians and/or quite high genetic homogeneity in our population. Five individuals (*GJB2* (+/−) subgroup), amounting to 3.4 % of unrelated affected participants were found to be heterozygous carriers of one recessive mutation. This result fits into the 95 % CI of *GJB2* gene mutation carrier frequency estimated in our population and possibly represents only carrier status.

Second goal of our study was to assess the burden of *DFNB1*-related early onset hearing loss in the Lithuanian population. Our group of participants represents the pure ethnic population due to the strict criteria for enrolment guaranteeing the uniqueness of this cohort: all 98 self-reported individuals indicated at least three generations of Lithuanian ethnicity and residency in the same ethno-linguistic region. Although a bigger ethnic population group would better reflect the current state of the amount of carriers of the *GJB2* gene mutation, but our results are nevertheless statistically reliable (p ≤ 0.05). The overall frequency of carriers of the *GJB2* gene mutation in the healthy group of our study amounted to 7.1 % (approx. - 1 in 14) allowing us to assess the *GJB2*-associated HL frequency in the Lithuanian population. It was estimated to be approximately 1 in 772 if the assortative marriages didn’t distort this value towards the higher edge. The results of the high frequency of carriers of the *GJB2* gene mutation in the ethnic Lithuanian groups of healthy participants demonstrate the significant *GJB2*-associated HL burden in our population.

Though the role of *GJB2* and *GJB6* gene alterations in the pathogenesis of sensorineural HL is undisputed, the structure of pathogenic changes identified in different populations is not uniform. The *GJB2* gene mutation c.35delG, p.(Gly12Valfs*2) (rs80338939) is the most frequent in Caucasian populations [25] and accounts for up to 70 % of mutated *GJB2* gene alleles. The c.167delT, p.(Leu56Argfs*26) (rs80338942) mutation is prevalent in the Ashkenazi Jewish population [26], and c.235delC, p.(Leu79Cysfs*3) (rs80338943) is the leading *GJB2* gene mutation in Eastern populations [27]. Splicing mutations (e.g. c. − 23 + 1G > A (rs80338940)) were found in some populations [28]. The most prevalent *GJB2* gene mutation in the affected group of participants of our study was c.35delG, p.(Gly12Valfs*2) (rs80338939). Its allele frequency (64.7 %) is consistent with many previously published studies in groups of affected individuals of Caucasian populations. The frequency of the c.35delG, p.(Gly12Valfs*2) (rs80338939) mutation in the healthy group in our study 1.0 % is less then described in other Caucasian populations where it can reach 3.2 % [29].

**Fig 6:**
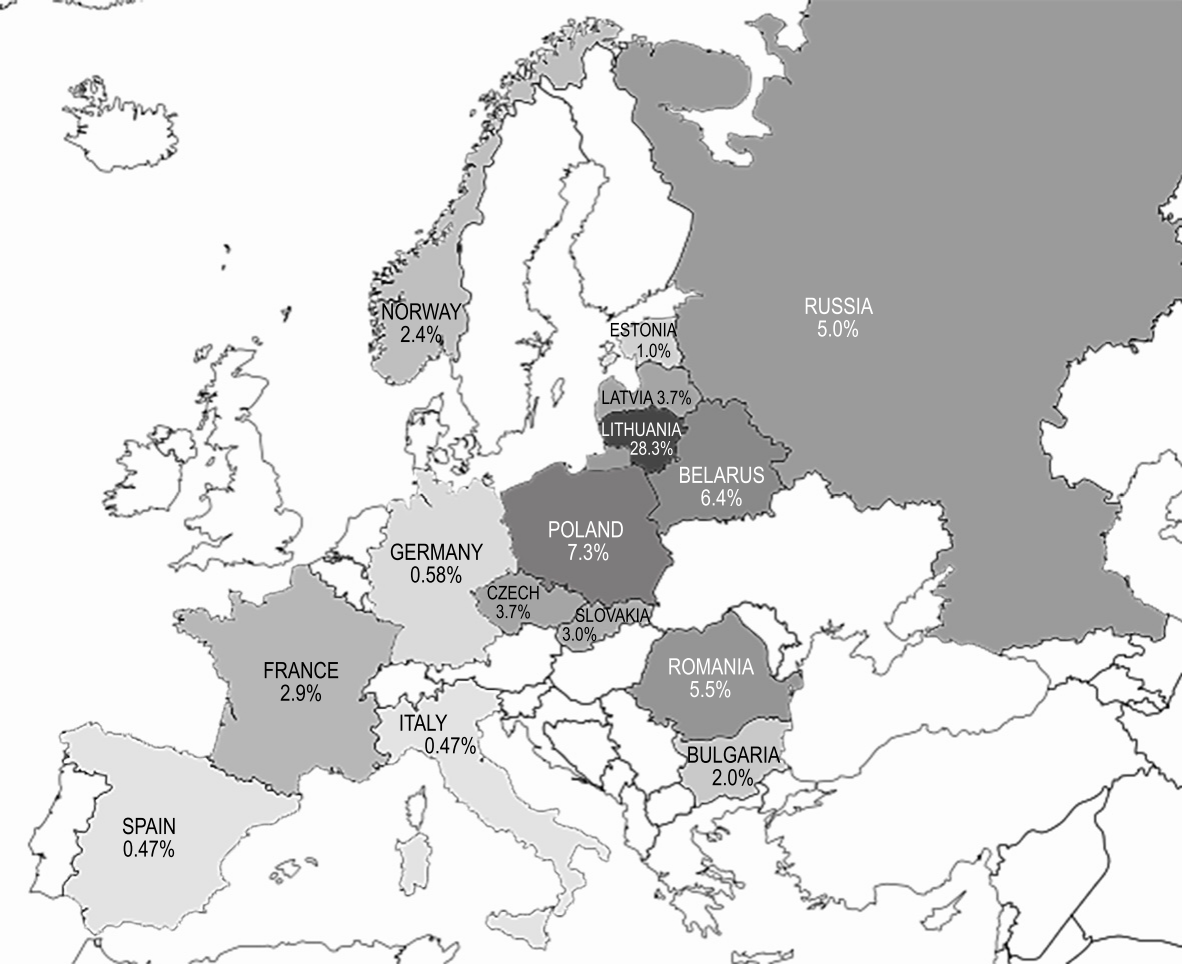
Allele frequencies of c.313_326del14, p.(Lys105Glyfs*5) (rs111033253) mutation in *GJB2*-positive groups of affected individuals in European populations (see references in *Discussion*). Adapted from Europe_political_chart available under Creative Commons Attribution-Share Alike 3.0 Unported and GNU Free Documentation Licenses

The c.313_326del14, p.(Lys105Glyfs*5) mutation (rs111033253), formerly called c.310del14, c.312del14, and c.314del14, truncates the *GJB2* gene and consequently interferes with the structural and functional integrity of connexons. To the best of our knowledge, this mutation has been identified previously in many European populations with a frequency of pathogenic alleles in the affected groups of participants from 0.5 % to 7.3 % (the highest allele frequency 7.3 % occurs in the Polish population (Fig. 6) [30–42]. The high frequency of the c.313_326del14, p.(Lys105Glyfs*5) (rs111033253) (28.32 % of pathogenic alleles) mutation in affected group of participants was an unexpected finding in our study. The c.[313_326del14];[313_326del14] genotype was found in 10.7 % of the *GJB2*-positive group of unrelated affected participants, suggesting not only a high frequency of carriers of this mutation in our population but also its possible origin in Lithuanian ancestors. The high frequency of carriers of the c.313_326del14, p.(Lys105Glyfs*5) (rs111033253) mutation in the entire Lithuanian population is supported by it being identified twice in the ethnic Lithuanian group of healthy participants (a frequency 2.0 % of carriers in the study group). The assumption that there is a high rate of carriers rate of this mutation in the Lithuanian population may also be supported by the coincidental finding of this mutation in the patient with syndromic type of hearing loss – Rogers syndrome [43] and in two affected participants having single *GJB2* gene mutation identified and possibly experiencing hearing loss of some other aetiology. The frequency of carriers of this mutation, 4.9x10^−4^, has been determined in the NHLBI Exome Sequencing Project in a group of European American descent, showing the extreme rarity of this mutation in the healthy population [44]. The mutation in a homozygous state has been found in 2 out of 12 *GJB2*-positive study participants (16.7 %) of Tatar ethnicity in Volga-Ural region of Russia [45]. These numbers are too low to make comprehensive conclusions, but homozygosity itself (with the exception of consanguinity) is a marker of a higher carrier rate in that particular population. In light of the close historical relationship between Lithuanians and Tatars during wars in 8^th^ – 14^th^ century this finding may provide a substantial basis for further analysis or multi-populational research of migration and assimilation processes in Eurasia. Recently literature review and *GJB2* mutations cluster analysis was published where Eastern European descent of the mutation was proposed [46]. Pilot genetic screening of hearing impairment in newborns from Grodno oblast (Belarus) revealed c.313_326del14, p.(Lys105Glyfs*5) (rs111033253) allele frequency 7 % and Polish origin was suggested [47]. From our analysis, we presume its Lithuanian descent. The relatively low frequency of the c.313_326del14, p.(Lys105Glyfs*5) (rs111033253) allele in the Latvian group of affected participants proves that the mutation emerged after the formation of the Baltic tribes. The higher frequencies of the c.313_326del14, p.(Lys105Glyfs*5) (rs111033253) allele amongst neighbouring countries (Poland and Grodno oblast of Belarus) may represent the spreading of the mutation due to close inter-relationships throughout the history of Lithuania.

Besides the undoubtedly disease-causing *GJB2* gene mutations, several changes have been disputed in scientific literature regarding their pathogenicity. The frequency of carriers of the c.101 T > C, p.Met34Thr (rs35887622) mutation was determined to be up to 6.5 % in the Caucasian population [29] and initially was reported as a polymorphism. The *in silico* computational analysis shows contradictory results (Table 2) but later publications explored this variation in functional analysis and concluded it to be pathogenic although with reduced penetrance [48, 49]. The results of the large UK population study recently published reaffirmed that this variant is associated with mild/moderate HL [50]. The *GJB2* mutation c.101 T > C (p.Met34Thr) (rs35887622) was underrepresented in the study group of affected individuals (allelic frequency 2.3 %) though its carrier rate in the healthy group of Lithuanian population is estimated to be 3.1 %. This finding may be explained by lower pathogenicity of the mutation leading to the later and milder manifestation of HL.

Another controversial *GJB2* gene change c.109G > A, p.(Val37Ile) (rs72474224) has also been debated. It was previously reported both as polymorphism and a pathogenic mutation [51, 52]. Although bioinformatics tools show some inconsistency in the evaluation of pathogenicity, this mutation is currently classified as pathogenic and associated with a mild to moderate phenotype [53]. The prevalence of c.109G > A, p.(Val37Ile) (rs72474224) was found to be higher in Eastern populations and this mutation was associated with the postnatal development of HL [54]. In the group of affected individuals we found one nuclear family with the AR mode of sensorineural HL inheritance possessing the aforementioned mutation leading to moderate HL.

The *GJB2* gene mutation c.379C > T, p.(Arg127Cys) (rs727503066) is considered to be a variant of uncertain significance in scientific literature [55] and according to *in silico* analysis (Table 2) but we classified it as a pathogenic mutation. Our decision was based on the observation that the mutation was identified in compound heterozygosity with c.35delG, p.(Gly12Valfs*2) (rs80338939) and with c.101 T > C, p.(Met34Thr) (rs35887622) mutations in two affected family members: father and daughter (the father having genotype c.[101 T > C];[379C > T] was not enrolled into the study because of the later onset of HL (over 5 years of age). This mutation was also described earlier in scientific literature: it has been identified in a compound heterozygous state with the c.35delG, p.(Gly12Valfs*2) (rs80338939) mutation in an affected individual [22].

*GJB6* gene mutations less contribute to the development of hearing loss but several mutations associated with HL have been described in scientific literature. Gross *DFNB1* locus deletions involving the *GJB6* gene – del(*GJB6*-D13S1830), del(*GJB6*-D13S1854), del(*DFNB1*-131 kb) and del(*DFNB1* > 920 kb) which encompass non-translated *GJB6* sequences essential for both *GJB6* and *GJB2* gene transcription have also been implicated in the pathogenesis of sensorineural HL [56]. In Lithuania we have not encountered any affected participant having *GJB6* gene point mutations or gross deletions indicating their rarity in our population. These results are similar to the previously published studies in other populations strengthening the evidence that *GJB6*-related non-syndromic hearing loss is extremely rare worldwide [57]. In the study group of healthy individuals of Lithuanian origin one carrier of the possibly pathogenic (evidence based on *in silico* prediction) *GJB6* gene mutation was identified.

The results of genotype – phenotype analysis show the significant impact of *GJB2* gene mutations on the development of early onset non-syndromic HL in affected group of Lithuanian origin. Our findings indicate that inactivating *GJB2* gene mutations were associated with a more severe phenotype than missense mutations – a finding compatible with previous publications [12] and the nature of the mutations. *GJB2* mutations also in general lead to more severe HL (OR 3.1, *p* = 0.003) with positive family history (OR 2.5, *p* = 0.013), compared with the non-*GJB2* aetiology of HL in Lithuanian population. Several studies have made comparisons of the characteristics of HL between *GJB2*-related and *GJB2*-negative groups of affected individuals, and statistically significant differences were determined in genealogy but not in disease severity or other HL characteristics [58]. These observations may be helpful in clinical settings to prognosticate the results of genetic testing and disease course to the patients with HL in the Lithuanian population.

## Conclusions

Analysis of the frequency of the pathogenic *GJB2* gene allele in the Lithuanian population revealed a high proportion of c. 313_326del14 mutations in the *GJB2*-positive group suggesting its possible origin in the ancestors of the Lithuanian population. The findings of high frequency of carriers of the *GJB2* gene mutation in the Lithuanian group of healthy participants led to the estimation of significant (1 in 772) *GJB2*-associated HL frequency in Lithuania. The findings of the study quantified the impact of mutations in the *DFNB1* genetic region to the development of HL in the Lithuanian population indicating the significant contribution of the *GJB2* gene mutations on the pathogenesis of the disorder. The results are useful for establishing the principles to predict the course of the disease in the patients with early onset of hearing loss.

### Availability of supporting data

The data sets supporting the results of this article are included within the article and its additional files.

## Additional files

Additional file 1:
**The conditions for PCR amplification of** ***GJB2*** **and** ***GJB6*** **genes coding sequences (primer sequences, annealing temperature, lengths of the amplicons).** (PDF 8 kb) Additional file 2:
**Dataset of the group of affected individuals (DEAFGEN project).** (PDF 191 kb) Additional file 3:
**Dataset of the group of ethnic Lithuanian population (LITGEN project).** (PDF 138 kb)

## Abbreviations

CHL: congenital hearing loss
DEAFGEN: the abbreviation of the project ‘The genomics of congenital/hereditary hearing loss: implication in disease pathogenesis, influence to phenotypic expression and treatment efficiency’
*DFNB1* locus: deafness, autosomal recessive 1 locus
EMQN: european molecular genetics quality network
*GJB2* and *GJB6* genes: gap junction protein, beta-2 and beta-6 genes
*GJB2*(+): *GJB2*-positive group of affected individuals (2 pathogenic *GJB2* gene mutations identified)
*GJB2*(+/−): 1 pathogenic *GJB2* gene mutation identified
*GJB2*(+): *GJB2*-negative group of affected individuals (no pathogenic *GJB2* gene mutation identified)
HL: hearing loss
I/I: subgroup of *GJB2*(+) group with 2 inactivating mutations identified
I/N: subgroup of *GJB2*(+) group with 1 inactivating and 1 non-inactivating mutations identified
LITGEN: the abbreviation of the project the ‘Genetic diversity of the population of Lithuania and changes of its genetic structure related with evolution and common diseases’
OMIM: online mendelian inheritance in man
OR: odds ratio
