# Supplementary material for: The high frequency of GJB2 gene mutation c.313_326del14 suggests its possible origin in ancestors of Lithuanian population
Source: BMC Genet. 2016 Feb 19;17:45. doi: 10.1186/s12863-016-0354-9 (PMC4761217; doi:10.1186/s12863-016-0354-9)
Supplement: Additional file 1: — The conditions for PCR amplification of GJB2 and GJB6 genes coding sequences (primer sequences, annealing temperature, lengths of the amplicons). (PDF 8 kb) [file 12863_2016_354_MOESM1_ESM.pdf]

The conditions for PCR amplification of *GJB2* and *GJB6* genes coding sequences

Gene	Amplicon No	Primers	$T_{annealing}$	Fragment length
GJB2	1	F GGGTGTGGGGTGCGGTTAAA<br>R AGAAACGCCCGCTCCAGAAG	57	365
	2a	F CGTCTTTTCCAGAGCAAACCGC<br>R CATGTCTCCGGTAGGCCACG	57	335
	2b	F ACGTGGCCTACCGGAGACAT<br>R ACGGGTTGCCTCATCCCTCT	57	460
GJB6	5a	F AATATCACCGTGTCACTTTCCCA<br>R ATGATTCGGAAAAAGATGCTGC	56	517
	5b	F AAGCAGAAGGTTCGGATAGAGG<br>R CAGGTTGGTATTGCCTTCTGGAG	56	490
