# Supplementary material for: The high frequency of GJB2 gene mutation c.313_326del14 suggests its possible origin in ancestors of Lithuanian population
Source: BMC Genet. 2016 Feb 19;17:45. doi: 10.1186/s12863-016-0354-9 (PMC4761217; doi:10.1186/s12863-016-0354-9)
Supplement: Additional file 2: — Dataset of the group of affected individuals (DEAFGEN project). (PDF 191 kb) [file 12863_2016_354_MOESM2_ESM.pdf]

DATASET OF DEAFGEN PROJECT GROUP OF AFFECTED PARTICIPANTS

No	Gender	*GJB2* gene genotype	*GJB6* gene genotype
1	Male	c.[35delG];[35delG], p.[(Gly12Valfs*2)];[(Gly12Valfs*2)]	c.[=];[=], p.[(=)];[(=)]
2	Female	c.[35delG];[35delG], p.[(Gly12Valfs*2)];[(Gly12Valfs*2)]	c.[=];[=], p.[(=)];[(=)]
3	Female	c.[35delG];[35delG], p.[(Gly12Valfs*2)];[(Gly12Valfs*2)]	c.[=];[=], p.[(=)];[(=)]
4	Female	c.[35delG];[35delG], p.[(Gly12Valfs*2)];[(Gly12Valfs*2)]	c.[=];[=], p.[(=)];[(=)]
5	Female	c.[35delG];[35delG], p.[(Gly12Valfs*2)];[(Gly12Valfs*2)]	c.[=];[=], p.[(=)];[(=)]
6	Male	c.[35delG];[35delG], p.[(Gly12Valfs*2)];[(Gly12Valfs*2)]	c.[=];[=], p.[(=)];[(=)]
7	Male	c.[35delG];[35delG], p.[(Gly12Valfs*2)];[(Gly12Valfs*2)]	c.[=];[=], p.[(=)];[(=)]
8	Female	c.[35delG];[35delG], p.[(Gly12Valfs*2)];[(Gly12Valfs*2)]	c.[=];[=], p.[(=)];[(=)]
9	Male	c.[35delG];[35delG], p.[(Gly12Valfs*2)];[(Gly12Valfs*2)]	c.[=];[=], p.[(=)];[(=)]
10	Female	c.[35delG];[35delG], p.[(Gly12Valfs*2)];[(Gly12Valfs*2)]	c.[=];[=], p.[(=)];[(=)]
11	Female	c.[35delG];[35delG], p.[(Gly12Valfs*2)];[(Gly12Valfs*2)]	c.[=];[=], p.[(=)];[(=)]
12	Female	c.[35delG];[35delG], p.[(Gly12Valfs*2)];[(Gly12Valfs*2)]	c.[=];[=], p.[(=)];[(=)]
13	Male	c.[35delG];[35delG], p.[(Gly12Valfs*2)];[(Gly12Valfs*2)]	c.[=];[=], p.[(=)];[(=)]
14	Male	c.[35delG];[35delG], p.[(Gly12Valfs*2)];[(Gly12Valfs*2)]	c.[=];[=], p.[(=)];[(=)]
15	Male	c.[35delG];[35delG], p.[(Gly12Valfs*2)];[(Gly12Valfs*2)]	c.[=];[=], p.[(=)];[(=)]
16	Female	c.[35delG];[35delG], p.[(Gly12Valfs*2)];[(Gly12Valfs*2)]	c.[=];[=], p.[(=)];[(=)]
17	Male	c.[35delG];[35delG], p.[(Gly12Valfs*2)];[(Gly12Valfs*2)]	c.[=];[=], p.[(=)];[(=)]
18	Male	c.[35delG];[35delG], p.[(Gly12Valfs*2)];[(Gly12Valfs*2)]	c.[=];[=], p.[(=)];[(=)]
19	Male	c.[35delG];[35delG], p.[(Gly12Valfs*2)];[(Gly12Valfs*2)]	c.[=];[=], p.[(=)];[(=)]
20	Female	c.[35delG];[35delG], p.[(Gly12Valfs*2)];[(Gly12Valfs*2)]	c.[=];[=], p.[(=)];[(=)]
21	Female	c.[35delG];[35delG], p.[(Gly12Valfs*2)];[(Gly12Valfs*2)]	c.[=];[=], p.[(=)];[(=)]
22	Female	c.[35delG];[35delG], p.[(Gly12Valfs*2)];[(Gly12Valfs*2)]	c.[=];[=], p.[(=)];[(=)]

23	Male	c.[35delG];[35delG], p.[(Gly12Valfs*2)];[(Gly12Valfs*2)]	c.[=];[=], p.[(=)];[(=)]
24	Male	c.[35delG];[35delG], p.[(Gly12Valfs*2)];[(Gly12Valfs*2)]	c.[=];[=], p.[(=)];[(=)]
25	Male	c.[35delG];[35delG], p.[(Gly12Valfs*2)];[(Gly12Valfs*2)]	c.[=];[=], p.[(=)];[(=)]
26	Female	c.[35delG];[35delG], p.[(Gly12Valfs*2)];[(Gly12Valfs*2)]	c.[=];[=], p.[(=)];[(=)]
27	Male	c.[35delG];[35delG], p.[(Gly12Valfs*2)];[(Gly12Valfs*2)]	c.[=];[=], p.[(=)];[(=)]
28	Female	c.[35delG];[35delG], p.[(Gly12Valfs*2)];[(Gly12Valfs*2)]	c.[=];[=], p.[(=)];[(=)]
29	Female	c.[35delG];[35delG], p.[(Gly12Valfs*2)];[(Gly12Valfs*2)]	c.[=];[=], p.[(=)];[(=)]
30	Male	c.[35delG];[35delG], p.[(Gly12Valfs*2)];[(Gly12Valfs*2)]	c.[=];[=], p.[(=)];[(=)]
31	Male	c.[35delG];[35delG], p.[(Gly12Valfs*2)];[(Gly12Valfs*2)]	c.[=];[=], p.[(=)];[(=)]
32	Female	c.[35delG];[35delG], p.[(Gly12Valfs*2)];[(Gly12Valfs*2)]	c.[=];[=], p.[(=)];[(=)]
33	Female	c.[35delG];[35delG], p.[(Gly12Valfs*2)];[(Gly12Valfs*2)]	c.[=];[=], p.[(=)];[(=)]
34	Female	c.[35delG];[35delG], p.[(Gly12Valfs*2)];[(Gly12Valfs*2)]	c.[=];[=], p.[(=)];[(=)]
35	Male	c.[35delG];[35delG], p.[(Gly12Valfs*2)];[(Gly12Valfs*2)]	c.[=];[=], p.[(=)];[(=)]
36	Female	c.[35delG];[35delG], p.[(Gly12Valfs*2)];[(Gly12Valfs*2)]	c.[=];[=], p.[(=)];[(=)]
37	Male	c.[35delG];[35delG], p.[(Gly12Valfs*2)];[(Gly12Valfs*2)]	c.[=];[=], p.[(=)];[(=)]
38	Female	c.[35delG];[313_326del14], p.[(Gly12Valfs*2)];[(Lys105Glyfs*5)]	c.[=];[=], p.[(=)];[(=)]
39	Female	c.[35delG];[313_326del14], p.[(Gly12Valfs*2)];[(Lys105Glyfs*5)]	c.[=];[=], p.[(=)];[(=)]
40	Female	c.[35delG];[313_326del14], p.[(Gly12Valfs*2)];[(Lys105Glyfs*5)]	c.[=];[=], p.[(=)];[(=)]
41	Male	c.[35delG];[313_326del14], p.[(Gly12Valfs*2)];[(Lys105Glyfs*5)]	c.[=];[=], p.[(=)];[(=)]
42	Female	c.[35delG];[313_326del14], p.[(Gly12Valfs*2)];[(Lys105Glyfs*5)]	c.[=];[=], p.[(=)];[(=)]
43	Female	c.[35delG];[313_326del14], p.[(Gly12Valfs*2)];[(Lys105Glyfs*5)]	c.[=];[=], p.[(=)];[(=)]
44	Male	c.[35delG];[313_326del14], p.[(Gly12Valfs*2)];[(Lys105Glyfs*5)]	c.[=];[=], p.[(=)];[(=)]
45	Male	c.[35delG];[313_326del14], p.[(Gly12Valfs*2)];[(Lys105Glyfs*5)]	c.[=];[=], p.[(=)];[(=)]

46	Female	c.[35delG];[313_326del14], p.[(Gly12Valfs*2)];[(Lys105Glyfs*5)]	c.[=];[=], p.[(=)];[(=)]
47	Female	c.[35delG];[313_326del14], p.[(Gly12Valfs*2)];[(Lys105Glyfs*5)]	c.[=];[=], p.[(=)];[(=)]
48	Male	c.[35delG];[313_326del14], p.[(Gly12Valfs*2)];[(Lys105Glyfs*5)]	c.[=];[=], p.[(=)];[(=)]
49	Male	c.[35delG];[313_326del14], p.[(Gly12Valfs*2)];[(Lys105Glyfs*5)]	c.[=];[=], p.[(=)];[(=)]
50	Male	c.[35delG];[313_326del14], p.[(Gly12Valfs*2)];[(Lys105Glyfs*5)]	c.[=];[=], p.[(=)];[(=)]
51	Male	c.[35delG];[313_326del14], p.[(Gly12Valfs*2)];[(Lys105Glyfs*5)]	c.[=];[=], p.[(=)];[(=)]
52	Female	c.[35delG];[313_326del14], p.[(Gly12Valfs*2)];[(Lys105Glyfs*5)]	c.[=];[=], p.[(=)];[(=)]
53	Male	c.[35delG];[313_326del14], p.[(Gly12Valfs*2)];[(Lys105Glyfs*5)]	c.[=];[=], p.[(=)];[(=)]
54	Female	c.[35delG];[313_326del14], p.[(Gly12Valfs*2)];[(Lys105Glyfs*5)]	c.[=];[=], p.[(=)];[(=)]
55	Male	c.[35delG];[313_326del14], p.[(Gly12Valfs*2)];[(Lys105Glyfs*5)]	c.[=];[=], p.[(=)];[(=)]
56	Male	c.[35delG];[313_326del14], p.[(Gly12Valfs*2)];[(Lys105Glyfs*5)]	c.[=];[=], p.[(=)];[(=)]
57	Male	c.[35delG];[313_326del14], p.[(Gly12Valfs*2)];[(Lys105Glyfs*5)]	c.[=];[=], p.[(=)];[(=)]
58	Female	c.[35delG];[313_326del14], p.[(Gly12Valfs*2)];[(Lys105Glyfs*5)]	c.[=];[=], p.[(=)];[(=)]
59	Female	c.[35delG];[313_326del14], p.[(Gly12Valfs*2)];[(Lys105Glyfs*5)]	c.[=];[=], p.[(=)];[(=)]
60	Male	c.[35delG];[313_326del14], p.[(Gly12Valfs*2)];[(Lys105Glyfs*5)]	c.[=];[=], p.[(=)];[(=)]
61	Male	c.[35delG];[313_326del14], p.[(Gly12Valfs*2)];[(Lys105Glyfs*5)]	c.[=];[=], p.[(=)];[(=)]
62	Male	c.[35delG];[313_326del14], p.[(Gly12Valfs*2)];[(Lys105Glyfs*5)]	c.[=];[=], p.[(=)];[(=)]
63	Male	c.[35delG];[313_326del14], p.[(Gly12Valfs*2)];[(Lys105Glyfs*5)]	c.[=];[=], p.[(=)];[(=)]
64	Male	c.[35delG];[313_326del14], p.[(Gly12Valfs*2)];[(Lys105Glyfs*5)]	c.[=];[=], p.[(=)];[(=)]
65	Female	c.[35delG];[313_326del14], p.[(Gly12Valfs*2)];[(Lys105Glyfs*5)]	c.[=];[=], p.[(=)];[(=)]
66	Male	c.[313_326del14];[313_326del14], p.[(Lys105Glyfs*5)];[(Lys105Glyfs*5)]	c.[=];[=], p.[(=)];[(=)]
67	Female	c.[313_326del14];[313_326del14], p.[(Lys105Glyfs*5)];[(Lys105Glyfs*5)]	c.[=];[=], p.[(=)];[(=)]
68	Male	c.[313_326del14];[313_326del14], p.[(Lys105Glyfs*5)];[(Lys105Glyfs*5)]	c.[=];[=], p.[(=)];[(=)]

69	Female	c.[313_326del14];[313_326del14], p.[(Lys105Glyfs*5)];[(Lys105Glyfs*5)]	c.[=];[=], p.[(=)];[(=)]
70	Male	c.[313_326del14];[313_326del14], p.[(Lys105Glyfs*5)];[(Lys105Glyfs*5)]	c.[=];[=], p.[(=)];[(=)]
71	Female	c.[313_326del14];[313_326del14], p.[(Lys105Glyfs*5)];[(Lys105Glyfs*5)]	c.[=];[=], p.[(=)];[(=)]
72	Female	c.[313_326del14];[313_326del14], p.[(Lys105Glyfs*5)];[(Lys105Glyfs*5)]	c.[=];[=], p.[(=)];[(=)]
73	Male	c.[313_326del14];[313_326del14], p.[(Lys105Glyfs*5)];[(Lys105Glyfs*5)]	c.[=];[=], p.[(=)];[(=)]
74	Female	c.[313_326del14];[313_326del14], p.[(Lys105Glyfs*5)];[(Lys105Glyfs*5)]	c.[=];[=], p.[(=)];[(=)]
75	Female	c.[35delG];[269T>C], p.[(Gly12Valfs*2)];[(Leu90Pro)]	c.[=];[=], p.[(=)];[(=)]
76	Male	c.[35delG];[269T>C], p.[(Gly12Valfs*2)];[(Leu90Pro)]	c.[=];[=], p.[(=)];[(=)]
77	Male	c.[35delG];[269T>C], p.[(Gly12Valfs*2)];[(Leu90Pro)]	c.[=];[=], p.[(=)];[(=)]
78	Male	c.[35delG];[167delT], p.[(Gly12Valfs*2)];[(Leu56Argfs*26)]	c.[=];[=], p.[(=)];[(=)]
79	Female	c.[35delG];[109G>A], p.[(Gly12Valfs*2)];[(Val37Ile)]	c.[=];[=], p.[(=)];[(=)]
80	Female	c.[35delG];[379C>T], p.[(Gly12Valfs*2)];[(Arg127Cys)]	c.[=];[=], p.[(=)];[(=)]
81	Male	c.[35delG];[101T>C], p.[(Gly12Valfs*2)];[(Met34Thr)]	c.[=];[=], p.[(=)];[(=)]
82	Female	c.[35delG];[101T>C], p.[(Gly12Valfs*2)];[(Met34Thr)]	c.[=];[=], p.[(=)];[(=)]
83	Male	c.[269T>C];[313_326del14], p.[(Leu90Pro)];[(Lys105Glyfs*5)]	c.[=];[=], p.[(=)];[(=)]
84	Female	c.[101T>C];[313_326del14], p.[(Met34Thr)];[(Lys105Glyfs*5)]	c.[=];[=], p.[(=)];[(=)]
85	Male	c.[101T>C];[=], p.[(Met34Thr)];[(=)]	c.[=];[=], p.[(=)];[(=)]
86	Female	c.[167delT];[=], p.[(Leu56Argfs*26)];[(=)]	c.[=];[=], p.[(=)];[(=)]
87	Male	c.[313_326del14];[=], p.[(Lys105Glyfs*5)];[(=)]	c.[=];[=], p.[(=)];[(=)]
88	Male	c.[35delG];[=], p.[(Gly12Valfs*2)];[(=)]	c.[=];[=], p.[(=)];[(=)]
89	Female	c.[35delG];[=], p.[(Gly12Valfs*2)];[(=)]	c.[=];[=], p.[(=)];[(=)]
90	Female	c.[=];[=], p.[(=)];[(=)]	c.[=];[=], p.[(=)];[(=)]
91	Male	c.[=];[=], p.[(=)];[(=)]	c.[=];[=], p.[(=)];[(=)]
92	Male	c.[=];[=], p.[(=)];[(=)]	c.[=];[=], p.[(=)];[(=)]
93	Female	c.[=];[=], p.[(=)];[(=)]	c.[=];[=], p.[(=)];[(=)]
94	Female	c.[=];[=], p.[(=)];[(=)]	c.[=];[=], p.[(=)];[(=)]
95	Female	c.[=];[=], p.[(=)];[(=)]	c.[=];[=], p.[(=)];[(=)]
96	Male	c.[=];[=], p.[(=)];[(=)]	c.[=];[=], p.[(=)];[(=)]

97	Female	c.[=];[=], p.[(=)];[(=)]	c.[=];[=], p.[(=)];[(=)]
98	Female	c.[=];[=], p.[(=)];[(=)]	c.[=];[=], p.[(=)];[(=)]
99	Male	c.[=];[=], p.[(=)];[(=)]	c.[=];[=], p.[(=)];[(=)]
100	Female	c.[=];[=], p.[(=)];[(=)]	c.[=];[=], p.[(=)];[(=)]
101	Male	c.[=];[=], p.[(=)];[(=)]	c.[=];[=], p.[(=)];[(=)]
102	Female	c.[=];[=], p.[(=)];[(=)]	c.[=];[=], p.[(=)];[(=)]
103	Female	c.[=];[=], p.[(=)];[(=)]	c.[=];[=], p.[(=)];[(=)]
104	Female	c.[=];[=], p.[(=)];[(=)]	c.[=];[=], p.[(=)];[(=)]
105	Female	c.[=];[=], p.[(=)];[(=)]	c.[=];[=], p.[(=)];[(=)]
106	Female	c.[=];[=], p.[(=)];[(=)]	c.[=];[=], p.[(=)];[(=)]
107	Female	c.[=];[=], p.[(=)];[(=)]	c.[=];[=], p.[(=)];[(=)]
108	Male	c.[=];[=], p.[(=)];[(=)]	c.[=];[=], p.[(=)];[(=)]
109	Female	c.[=];[=], p.[(=)];[(=)]	c.[=];[=], p.[(=)];[(=)]
110	Female	c.[=];[=], p.[(=)];[(=)]	c.[=];[=], p.[(=)];[(=)]
111	Female	c.[=];[=], p.[(=)];[(=)]	c.[=];[=], p.[(=)];[(=)]
112	Female	c.[=];[=], p.[(=)];[(=)]	c.[=];[=], p.[(=)];[(=)]
113	Male	c.[=];[=], p.[(=)];[(=)]	c.[=];[=], p.[(=)];[(=)]
114	Male	c.[=];[=], p.[(=)];[(=)]	c.[=];[=], p.[(=)];[(=)]
115	Female	c.[=];[=], p.[(=)];[(=)]	c.[=];[=], p.[(=)];[(=)]
116	Female	c.[=];[=], p.[(=)];[(=)]	c.[=];[=], p.[(=)];[(=)]
117	Male	c.[=];[=], p.[(=)];[(=)]	c.[=];[=], p.[(=)];[(=)]
118	Female	c.[=];[=], p.[(=)];[(=)]	c.[=];[=], p.[(=)];[(=)]
119	Female	c.[=];[=], p.[(=)];[(=)]	c.[=];[=], p.[(=)];[(=)]
120	Male	c.[=];[=], p.[(=)];[(=)]	c.[=];[=], p.[(=)];[(=)]
121	Male	c.[=];[=], p.[(=)];[(=)]	c.[=];[=], p.[(=)];[(=)]
122	Male	c.[=];[=], p.[(=)];[(=)]	c.[=];[=], p.[(=)];[(=)]
123	Female	c.[=];[=], p.[(=)];[(=)]	c.[=];[=], p.[(=)];[(=)]
124	Male	c.[=];[=], p.[(=)];[(=)]	c.[=];[=], p.[(=)];[(=)]
125	Male	c.[=];[=], p.[(=)];[(=)]	c.[=];[=], p.[(=)];[(=)]
126	Female	c.[=];[=], p.[(=)];[(=)]	c.[=];[=], p.[(=)];[(=)]
127	Female	c.[=];[=], p.[(=)];[(=)]	c.[=];[=], p.[(=)];[(=)]
128	Male	c.[=];[=], p.[(=)];[(=)]	c.[=];[=], p.[(=)];[(=)]
129	Male	c.[=];[=], p.[(=)];[(=)]	c.[=];[=], p.[(=)];[(=)]
130	Female	c.[=];[=], p.[(=)];[(=)]	c.[=];[=], p.[(=)];[(=)]
131	Female	c.[=];[=], p.[(=)];[(=)]	c.[=];[=], p.[(=)];[(=)]
132	Male	c.[=];[=], p.[(=)];[(=)]	c.[=];[=], p.[(=)];[(=)]
133	Male	c.[=];[=], p.[(=)];[(=)]	c.[=];[=], p.[(=)];[(=)]
134	Male	c.[=];[=], p.[(=)];[(=)]	c.[=];[=], p.[(=)];[(=)]
135	Female	c.[=];[=], p.[(=)];[(=)]	c.[=];[=], p.[(=)];[(=)]
136	Male	c.[=];[=], p.[(=)];[(=)]	c.[=];[=], p.[(=)];[(=)]
137	Female	c.[=];[=], p.[(=)];[(=)]	c.[=];[=], p.[(=)];[(=)]

138	Male	c.[=];[=], p.[(=)];[(=)]	c.[=];[=], p.[(=)];[(=)]
139	Male	c.[=];[=], p.[(=)];[(=)]	c.[=];[=], p.[(=)];[(=)]
140	Male	c.[=];[=], p.[(=)];[(=)]	c.[=];[=], p.[(=)];[(=)]
141	Male	c.[=];[=], p.[(=)];[(=)]	c.[=];[=], p.[(=)];[(=)]
142	Male	c.[=];[=], p.[(=)];[(=)]	c.[=];[=], p.[(=)];[(=)]
143	Female	c.[=];[=], p.[(=)];[(=)]	c.[=];[=], p.[(=)];[(=)]
144	Male	c.[=];[=], p.[(=)];[(=)]	c.[=];[=], p.[(=)];[(=)]
145	Male	c.[=];[=], p.[(=)];[(=)]	c.[=];[=], p.[(=)];[(=)]
146	Female	c.[=];[=], p.[(=)];[(=)]	c.[=];[=], p.[(=)];[(=)]
