# Supplementary material for: The high frequency of GJB2 gene mutation c.313_326del14 suggests its possible origin in ancestors of Lithuanian population
Source: BMC Genet. 2016 Feb 19;17:45. doi: 10.1186/s12863-016-0354-9 (PMC4761217; doi:10.1186/s12863-016-0354-9)
Supplement: Additional file 3: — Dataset of the group of ethnic Lithuanian population (LITGEN project). (PDF 138 kb) [file 12863_2016_354_MOESM3_ESM.pdf]

DATASET OF THE LITGEN PROJECT GROUP OF ETHNIC LITHUANIAN POPULATION

No.	Gender	*GJB2* gene genotype and SNP identification number*	*GJB6* gene genotype and SNP identification number*
1	Female	c.[35delG];[=]<br>p.[(Gly12Valfs*2)];[(=)]<br>rs80338939	c.[=];[=], p.[(=)];[(=)]
2	Female	c.[101T>C];[=]<br>p.[(Met34Arg)];[(=)]<br>rs35887622	c.[=];[=], p.[(=)];[(=)]
3	Female	c.[101T>C];[=]<br>p.[(Met34Arg)];[(=)]<br>rs35887622	c.[=];[=], p.[(=)];[(=)]
4	Female	c.[101T>C];[=]<br>p.[(Met34Arg)];[(=)]<br>rs35887622	c.[=];[=], p.[(=)];[(=)]
5	Male	c.[313_326delAAGTTCATCAAGGG];[=]<br>p.[(Lys105Glyfs*5)];[(=)]<br>rs111033253	c.[=];[=], p.[(=)];[(=)]
6	Female	c.[313_326delAAGTTCATCAAGGG];[=]<br>p.[(Lys105Glyfs*5)];[(=)]<br>rs111033253	c.[=];[=], p.[(=)];[(=)]
7	Female	c.[206delT];[=]<br>p.[(Phe69Serfs*13)];[(=)]	c.[=];[=], p.[(=)];[(=)]
8	Male	c.[=];[=], p.[(=)];[(=)]	c.[428G>A];[(=)]<br>p.[(Arg143Gln)];[(=)]<br>rs201783640
9	Male	c.[=];[=], p.[(=)];[(=)]	c.[=];[=], p.[(=)];[(=)]
10	Female	c.[=];[=], p.[(=)];[(=)]	c.[=];[=], p.[(=)];[(=)]
11	Male	c.[=];[=], p.[(=)];[(=)]	c.[=];[=], p.[(=)];[(=)]
12	Female	c.[=];[=], p.[(=)];[(=)]	c.[=];[=], p.[(=)];[(=)]
13	Female	c.[=];[=], p.[(=)];[(=)]	c.[=];[=], p.[(=)];[(=)]
14	Male	c.[=];[=], p.[(=)];[(=)]	c.[=];[=], p.[(=)];[(=)]
15	Male	c.[=];[=], p.[(=)];[(=)]	c.[=];[=], p.[(=)];[(=)]
16	Female	c.[=];[=], p.[(=)];[(=)]	c.[=];[=], p.[(=)];[(=)]
17	Male	c.[=];[=], p.[(=)];[(=)]	c.[=];[=], p.[(=)];[(=)]
18	Female	c.[=];[=], p.[(=)];[(=)]	c.[=];[=], p.[(=)];[(=)]
19	Female	c.[=];[=], p.[(=)];[(=)]	c.[=];[=], p.[(=)];[(=)]
20	Male	c.[=];[=], p.[(=)];[(=)]	c.[=];[=], p.[(=)];[(=)]
21	Male	c.[=];[=], p.[(=)];[(=)]	c.[=];[=], p.[(=)];[(=)]
22	Female	c.[=];[=], p.[(=)];[(=)]	c.[=];[=], p.[(=)];[(=)]
23	Male	c.[=];[=], p.[(=)];[(=)]	c.[=];[=], p.[(=)];[(=)]
24	Female	c.[=];[=], p.[(=)];[(=)]	c.[=];[=], p.[(=)];[(=)]
25	Male	c.[=];[=], p.[(=)];[(=)]	c.[=];[=], p.[(=)];[(=)]
26	Male	c.[=];[=], p.[(=)];[(=)]	c.[=];[=], p.[(=)];[(=)]
27	Female	c.[=];[=], p.[(=)];[(=)]	c.[=];[=], p.[(=)];[(=)]
28	Male	c.[=];[=], p.[(=)];[(=)]	c.[=];[=], p.[(=)];[(=)]
29	Female	c.[=];[=], p.[(=)];[(=)]	c.[=];[=], p.[(=)];[(=)]
30	Male	c.[=];[=], p.[(=)];[(=)]	c.[=];[=], p.[(=)];[(=)]
31	Male	c.[=];[=], p.[(=)];[(=)]	c.[=];[=], p.[(=)];[(=)]

32	Male	c.[=];[=], p.[(=)];[(=)]	c.[=];[=], p.[(=)];[(=)]
33	Female	c.[=];[=], p.[(=)];[(=)]	c.[=];[=], p.[(=)];[(=)]
34	Male	c.[=];[=], p.[(=)];[(=)]	c.[=];[=], p.[(=)];[(=)]
35	Male	c.[=];[=], p.[(=)];[(=)]	c.[=];[=], p.[(=)];[(=)]
36	Female	c.[=];[=], p.[(=)];[(=)]	c.[=];[=], p.[(=)];[(=)]
38	Female	c.[=];[=], p.[(=)];[(=)]	c.[=];[=], p.[(=)];[(=)]
39	Female	c.[=];[=], p.[(=)];[(=)]	c.[=];[=], p.[(=)];[(=)]
40	Male	c.[=];[=], p.[(=)];[(=)]	c.[=];[=], p.[(=)];[(=)]
41	Female	c.[=];[=], p.[(=)];[(=)]	c.[=];[=], p.[(=)];[(=)]
42	Female	c.[=];[=], p.[(=)];[(=)]	c.[=];[=], p.[(=)];[(=)]
43	Male	c.[=];[=], p.[(=)];[(=)]	c.[=];[=], p.[(=)];[(=)]
44	Male	c.[=];[=], p.[(=)];[(=)]	c.[=];[=], p.[(=)];[(=)]
45	Female	c.[=];[=], p.[(=)];[(=)]	c.[=];[=], p.[(=)];[(=)]
46	Female	c.[=];[=], p.[(=)];[(=)]	c.[=];[=], p.[(=)];[(=)]
47	Female	c.[=];[=], p.[(=)];[(=)]	c.[=];[=], p.[(=)];[(=)]
48	Female	c.[=];[=], p.[(=)];[(=)]	c.[=];[=], p.[(=)];[(=)]
49	Male	c.[=];[=], p.[(=)];[(=)]	c.[=];[=], p.[(=)];[(=)]
50	Male	c.[=];[=], p.[(=)];[(=)]	c.[=];[=], p.[(=)];[(=)]
51	Female	c.[=];[=], p.[(=)];[(=)]	c.[=];[=], p.[(=)];[(=)]
52	Male	c.[=];[=], p.[(=)];[(=)]	c.[=];[=], p.[(=)];[(=)]
53	Male	c.[=];[=], p.[(=)];[(=)]	c.[=];[=], p.[(=)];[(=)]
54	Male	c.[=];[=], p.[(=)];[(=)]	c.[=];[=], p.[(=)];[(=)]
55	Female	c.[=];[=], p.[(=)];[(=)]	c.[=];[=], p.[(=)];[(=)]
56	Male	c.[=];[=], p.[(=)];[(=)]	c.[=];[=], p.[(=)];[(=)]
57	Male	c.[=];[=], p.[(=)];[(=)]	c.[=];[=], p.[(=)];[(=)]
58	Female	c.[=];[=], p.[(=)];[(=)]	c.[=];[=], p.[(=)];[(=)]
59	Male	c.[=];[=], p.[(=)];[(=)]	c.[=];[=], p.[(=)];[(=)]
60	Male	c.[=];[=], p.[(=)];[(=)]	c.[=];[=], p.[(=)];[(=)]
61	Male	c.[=];[=], p.[(=)];[(=)]	c.[=];[=], p.[(=)];[(=)]
62	Male	c.[=];[=], p.[(=)];[(=)]	c.[=];[=], p.[(=)];[(=)]
63	Female	c.[=];[=], p.[(=)];[(=)]	c.[=];[=], p.[(=)];[(=)]
64	Female	c.[=];[=], p.[(=)];[(=)]	c.[=];[=], p.[(=)];[(=)]
65	Male	c.[=];[=], p.[(=)];[(=)]	c.[=];[=], p.[(=)];[(=)]
66	Female	c.[=];[=], p.[(=)];[(=)]	c.[=];[=], p.[(=)];[(=)]
67	Female	c.[=];[=], p.[(=)];[(=)]	c.[=];[=], p.[(=)];[(=)]
68	Male	c.[=];[=], p.[(=)];[(=)]	c.[=];[=], p.[(=)];[(=)]
69	Male	c.[=];[=], p.[(=)];[(=)]	c.[=];[=], p.[(=)];[(=)]
70	Male	c.[=];[=], p.[(=)];[(=)]	c.[=];[=], p.[(=)];[(=)]
71	Male	c.[=];[=], p.[(=)];[(=)]	c.[=];[=], p.[(=)];[(=)]
72	Female	c.[=];[=], p.[(=)];[(=)]	c.[=];[=], p.[(=)];[(=)]
73	Female	c.[=];[=], p.[(=)];[(=)]	c.[=];[=], p.[(=)];[(=)]
74	Male	c.[=];[=], p.[(=)];[(=)]	c.[=];[=], p.[(=)];[(=)]
75	Female	c.[=];[=], p.[(=)];[(=)]	c.[=];[=], p.[(=)];[(=)]
76	Male	c.[=];[=], p.[(=)];[(=)]	c.[=];[=], p.[(=)];[(=)]
77	Female	c.[=];[=], p.[(=)];[(=)]	c.[=];[=], p.[(=)];[(=)]
78	Male	c.[=];[=], p.[(=)];[(=)]	c.[=];[=], p.[(=)];[(=)]
79	Male	c.[=];[=], p.[(=)];[(=)]	c.[=];[=], p.[(=)];[(=)]
80	Female	c.[=];[=], p.[(=)];[(=)]	c.[=];[=], p.[(=)];[(=)]
81	Male	c.[=];[=], p.[(=)];[(=)]	c.[=];[=], p.[(=)];[(=)]
82	Female	c.[=];[=], p.[(=)];[(=)]	c.[=];[=], p.[(=)];[(=)]
83	Female	c.[=];[=], p.[(=)];[(=)]	c.[=];[=], p.[(=)];[(=)]
84	Female	c.[=];[=], p.[(=)];[(=)]	c.[=];[=], p.[(=)];[(=)]
85	Male	c.[=];[=], p.[(=)];[(=)]	c.[=];[=], p.[(=)];[(=)]

86	Female	c.[=];[=], p.[(=)];[(=)]	c.[=];[=], p.[(=)];[(=)]
87	Male	c.[=];[=], p.[(=)];[(=)]	c.[=];[=], p.[(=)];[(=)]
88	Female	c.[=];[=], p.[(=)];[(=)]	c.[=];[=], p.[(=)];[(=)]
89	Female	c.[=];[=], p.[(=)];[(=)]	c.[=];[=], p.[(=)];[(=)]
90	Male	c.[=];[=], p.[(=)];[(=)]	c.[=];[=], p.[(=)];[(=)]
91	Male	c.[=];[=], p.[(=)];[(=)]	c.[=];[=], p.[(=)];[(=)]
92	Female	c.[=];[=], p.[(=)];[(=)]	c.[=];[=], p.[(=)];[(=)]
93	Male	c.[=];[=], p.[(=)];[(=)]	c.[=];[=], p.[(=)];[(=)]
94	Female	c.[=];[=], p.[(=)];[(=)]	c.[=];[=], p.[(=)];[(=)]
95	Male	c.[=];[=], p.[(=)];[(=)]	c.[=];[=], p.[(=)];[(=)]
96	Female	c.[=];[=], p.[(=)];[(=)]	c.[=];[=], p.[(=)];[(=)]
97	Female	c.[=];[=], p.[(=)];[(=)]	c.[=];[=], p.[(=)];[(=)]
98	Female	c.[=];[=], p.[(=)];[(=)]	c.[=];[=], p.[(=)];[(=)]

*According to NCBI dbSNP database
